# Dietary Phytochemicals Promote Health by Enhancing Antioxidant Defence in a Pig Model

**DOI:** 10.3390/nu9070758

**Published:** 2017-07-14

**Authors:** Sophie N. B. Selby-Pham, Jeremy J. Cottrell, Frank R. Dunshea, Ken Ng, Louise E. Bennett, Kate S. Howell

**Affiliations:** 1 Faculty of Veterinary and Agricultural, The University of Melbourne, Parkville, VIC 3010, Australia; s.selbypham@gmail.com (S.N.B.S.-P.); jcottrell@unimelb.edu.au (J.J.C.); fdunshea@unimelb.edu.au (F.R.D.); ngkf@unimelb.edu.au (K.N.); 2 CSIRO Agriculture and Food, 671 Sneydes Road, Werribee, VIC 3010, Australia; louise.bennett1@monash.edu

**Keywords:** hydrogen peroxide, reactive oxygen species, plant extracts, red cabbage, grape, glutathione peroxide, total antioxidant capacity, porcine, piglet, Landrace

## Abstract

Phytochemical-rich diets are protective against chronic diseases and mediate their protective effect by regulation of oxidative stress (OS). However, it is proposed that under some circumstances, phytochemicals can promote production of reactive oxygen species (ROS) in vitro, which might drive OS-mediated signalling. Here, we investigated the effects of administering single doses of extracts of red cabbage and grape skin to pigs. Blood samples taken at baseline and 30 min intervals for 4 hours following intake were analyzed by measures of antioxidant status in plasma, including Trolox equivalent antioxidant capacity (TEAC) and glutathione peroxidase (GPx) activity. In addition, dose-dependent production of hydrogen peroxide (H_2_O_2_) by the same extracts was measured in untreated commercial pig plasma in vitro. Plasma from treated pigs showed extract dose-dependent increases in non-enzymatic (plasma TEAC) and enzymatic (GPx) antioxidant capacities. Similarly, extract dose-dependent increases in H_2_O_2_ were observed in commercial pig plasma in vitro. The antioxidant responses to extracts by treated pigs were highly correlated with their respective yields of H_2_O_2_ production in vitro. These results support that dietary phytochemicals regulate OS via direct and indirect antioxidant mechanisms. The latter may be attributed to the ability to produce H_2_O_2_ and to thereby stimulate cellular antioxidant defence systems.

## 1. Introduction

A phytochemical-rich diet is strongly associated with reducing the risk of chronic diseases including cancer [1], cardiovascular [2], and neurodegenerative diseases [3]. The health benefits of dietary phytochemicals have been attributed to their ability to mitigate oxidative stress and inflammation (OSI), which is associated with normal metabolism [4,5] but is also involved in the onset of chronic diseases [6]. Production of reactive oxygen species (ROS) occurs under normal conditions in cells, the main source from by-products of the electron transport chains [7]. Uncontrolled ROS can lead to OSI and unregulated OSI can result in molecular and cellular damage which in turn leads to an increased risk of chronic diseases [8]. However, OSI is an important defence mechanism of the body against infections and injuries [9]. Therefore, transient peaks or optimal steady state levels of ROS in the body are likely involved in maintaining good health and reducing the risk of disease [10].

It was believed that dietary phytochemicals exert protection via direct scavenging of ROS, as observed in many in vitro studies [11,12,13]. However, this concept has been challenged as the concentrations of phytochemicals in human plasma in vivo after consumption of phytochemicals are much lower (in the nM to low µM range) compared to concentrations used in the in vitro studies (in the low µM to mM range) [14,15]. There are clearly discrepancies between the studies of these mechanisms in the whole organism.

The health benefits of dietary phytochemicals are thought to be attributed to their ability to generate electrophilic or chemical stress signals, which trigger the cellular defence system to protect against molecular damage and subsequent chronic diseases [16,17,18,19]. The cellular antioxidant defence is made up of a non-enzymatic, inducible enzymatic defence and the DNA repair systems [20]. The non-enzymatic defence includes antioxidant molecules such as vitamin C, vitamin E, uric acid, glutathione, and thioredoxin that directly scavenge ROS and metal-chelating proteins such as transferrin, coeruloplasmin, and metallothionein that prevent ROS formation via controlling the level of pro-oxidative free metal ions [20]. The enzymatic antioxidant defence includes several pathways that remove ROS through enzymatic reactions. For example, superoxide dismutase converts superoxide anions into hydrogen peroxide (H_2_O_2_), which is subsequently transformed by catalase into oxygen and water or by glutathione peroxidase (GPx) into water [20]. The reduction of H_2_O_2_ by GPx consumes the reduced form of glutathione and generates the oxidised form, which can be recycled by glutathione reductase to restore the glutathione pool [20]. 

Dietary phytochemicals have been associated with increasing the levels of both non-enzymatic and enzymatic antioxidant defence in animal [21,22,23,24,25,26] and human dietary intervention studies [27,28,29,30]. Consumption of phytochemical-rich diets increased the expression of genes associated with DNA repair, immune, and inflammatory responses in humans [10,31,32,33]. The varied roles that dietary phytochemicals may play in the whole organism are complex, perhaps overlapping and have not been fully elucidated. The ability of dietary phytochemicals to generate stress signals can be related to their ability to produce ROS, in particular H_2_O_2_ [34]. Phytochemicals have been reported to produce H_2_O_2_ in cell culture media, which was potentially responsible for their cytotoxic effects in cell culture studies [35,36,37,38]. However, no research has been done on the ability of phytochemicals to produce H_2_O_2_ in plasma. Understanding this pro-oxidant action will provide information about how the phytochemicals can stimulate ROS-induced cellular antioxidant defence to provide protective effects against OSI.

Absorption of phytochemicals into circulation and uptake by target cells are essential for phytochemicals to exert biological effects [39]. As phytochemicals are recognised by the human body as xenobiotics, their presence in the human body is transient [40] and influenced by their physicochemical properties. Recently, we have developed the phytochemical absorption prediction (PCAP) model, allowing direct calculation of the time required for phytochemicals to reach their maximal plasma concentrations (T_max_) after oral consumption, based on their molecular mass and lipophilicity descriptor log P [41]. Further, a liquid chromatography mass spectrometry (LC-MS) method has been developed to characterise T_max_ ranges of phytochemical mixtures based on molecular mass and log P [42]. Here, we extend this modelling to dietary intervention in pigs, an animal model with physiological and anatomical similarities to the digestive tract of humans [43].

Phytochemicals across a broad range of chemical classes have been shown to impart positive health benefits [3,40]. Grape products and *Brassica* vegetables are among the most widely studied for their antioxidant capacity and protection against chronic diseases [44,45]. Grape skin contains predominately polyphenols including anthocyanidins, phenolic acids, and stilbenes [44], whilst red cabbage (a member of the *Brassica* vegetables) contains a wider variety of phytochemicals including polyphenols (anthocyanidins, phenolic acids), glucosinolates, and vitamins [46].

The aim of this study was to use a pig model to establish the absorption kinetics of phytochemical extracts from red cabbage and grape skin and to examine their effects on two measures of antioxidant status (plasma total antioxidant capacity and plasma GPx activity). Direct induction of the pro-oxidant effects of the plant extracts in pig plasma was measured by H_2_O_2_ production in pig plasma when exposed to the plant extracts in vitro. This study provides both in vitro and ex vivo evidence to support that one of the likely modes of action by phytochemicals is to induce H_2_O_2_ in plasma and to thereby initiate protective action by enzymatic and non-enzymatic cellular defences.

## 2. Materials and Methods

### 2.1. Materials

All chemicals including gallic acid, Folin-Ciocalteu reagent, sodium carbonate (Na_2_CO_3_), hydrogen peroxide (H_2_O_2_), sulfuric acid (H_2_SO_4_), xylenol orange, Iron(II) sulphate (FeSO_4_), butylated hydroxytoluene (BHT), tris(hydroxymethyl)aminomethane (Tris), glycine, citrate, urea, hydrochloric acid (HCl), Trolox, bathocuproinedisulfonic acid sodium salt (BCS), copper(II) chloride (CuCl_2_), methanol, formic acid, acetonitrile, l-histidine, (*S*)-dihydroorotate, shikimate, 4-pyridoxate, 3-hydroxybenzyl alcohol, 2,5-dihydroxybenzoate, 3-hydroxybenzaldehyde, trans-cinnamate, estradiol-17α, deoxycholate, retinoate, oleic acid, and heptadecanoate were of analytical grade and from Sigma-Aldrich (St. Louis, MO, USA). 96 well plates were from Greiner UV-Star (Greiner Bio-One, Frickenhausen, Germany).

Tris-glycine-urea buffer pH 7 contained 0.086 M Tris, 0.09 M glycine, 4 mM citrate, and 8 M urea, adjusted to pH 7 using 2 M HCl. Ferrous ion oxidation-xylenol orange (FOX) reagent contained 25 mM H_2_SO_4_ containing 0.1 mM xylenol orange, 0.25 mM FeSO_4_, and 4 mM BHT in 90% methanol.

### 2.2. Preparation of Plant Extracts

Grape skin extract was obtained from Tarac Technologies (Nuriootpa, South Australia, Australia). The extract was freeze-dried (Virtis Genesis 35EL, SP Scientific, Warmister, PA, USA) and stored with a small head space with desiccant at −18 °C.

Red cabbage extract was produced by the following process. Fresh red cabbage was purchased from a local retailer (Coles supermarket, Werribee, Victoria, Australia). Edible parts of the red cabbage were washed and blended in a food processor with water (1:2 ratio, *w*/*v*) before boiling by microwave heating at 800 W for 10 min. After cooling to ambient temperature, the mixture was ultrasonicated at 300 W for 11 min (Hielscher, Germany) before bag filtration (1 µm pore size, Sefar Filtration Inc., Depew, NY, USA). The filtrate was freeze-dried (Virtis Genesis 35EL, SP Scientific, Warmister, PA, USA) and stored with desiccant and low head space at −18 °C.

### 2.3. Total Phenolic Content of Plant Extracts

Total phenolic content of the plant extracts was quantified using the Folin-Ciocalteu assay [47]. In brief, 20 µL samples (blank, standard, or 2 mg/mL plant extract in 20% methanol) was added to 1 mL of 0.2 N Folin-Ciocalteu reagent and 180 µL of Milli-Q water and mixed for 15 s, and allowed to stand for 3 min before 800 µL of 7.5% Na_2_CO_3_ was added to the mixture. The mixture was shaken for 15 s followed by incubation at 37 °C for 1 h in the dark. The absorbance at 765 nm was measured using a Varioskan Flash microplate reader (Thermo Fisher Scientific, Waltham, MA, USA). The total phenolic content of plant extracts was reported as gallic acid equivalent (GAE) using a 7-point calibration curve of gallic acid standard with concentrations of 0–500 µg/mL in 20% methanol after blank subtraction. Total phenolic content of the plant extracts was 26.6 ± 1.5 mg GAE/g for red cabbage extract and 327.1 ± 13.9 mg GAE/g for grape skin extract. Analysis was performed in duplicate.

### 2.4. Prediction of Human Absorption Kinetics of Plant Extracts

Predicted human absorption kinetics, the ”functional fingerprints” of plant extracts, were determined using untargeted liquid chromatography mass spectrometry (LC-MS) profiling method [42] in combination with the PCAP model [41]. Untargeted LC-MS profiling analysis was performed using an Agilent 6520 quadrupole time-of-flight (QTOF) MS system (Agilent, Santa Clara, CA, USA) with a dual sprayer electrospray ionisation (ESI) source attached to the Agilent 1200 series high performance liquid chromatography (HPLC) system (Santa Clara, CA, USA) comprised of a vacuum degasser and binary pump with a thermostated auto-sampler and column oven. The MS was operated in positive or negative mode using the following conditions (positive/negative, respectively): nebulizer pressure 30/45 psi, gas flow-rate 10 L/min, gas temperature 300 °C, capillary voltage 4000/−3500 V, fragmentor 150, and skimmer 65 V. The instrument was operated in the extended dynamic range mode with data collected in the mass to change ratio (*m*/*z*) range of 70–1700. Chromatography was carried out using an Agilent Zorbax Eclipse XDB-C18, 2.1 × 100 mm, 1.8 µm column maintained at 40 °C (±1 °C) at a flow rate of 400 µL/min with a 20-min run time. A gradient LC method was used with mobile phases comprised of (A) 0.1% formic acid in deionized water and (B) 0.1% formic acid in acetonitrile. Gradient: A 5-min linear gradient from 5% to 30% mobile phase B, followed by 5-min gradient to 100% mobile phase B and then a 5-min hold, followed by a 5-min re-equilibration at 5% mobile phase B. Molecular feature extraction (MFE) was conducted using Agilent MassHunter Qualitative analysis (version B.07.00, Agilent) and MassHunter Profinder (version B.06.00, Agilent). Binning and alignment tolerances were set to: retention time: ±0.1% + 0.15 min; mass window: ±20 ppm + 2 mDa. Allowed ion species: H^+^, Na^+^, K^+^, NH_4_^+^, and neutral losses: H_2_O, H_3_PO_4_, CO_2_, C_6_H_12_O_6_. MFE was restricted to the 1000 largest features and 1–2 charge states. After elimination of the molecular features which were common in the two plant extracts (i.e., primary metabolites), the remaining molecular features represented the phytochemicals (i.e., secondary metabolites) of the plant extracts.

The lipophilicity descriptor log P was determined using a calibration curve of retention time and log P of twelve standards including l-histidine, (*S*)-dihydroorotate, shikimate, 4-pyridoxate, 3-hydroxybenzyl alcohol, 2,5-dihydroxybenzoate, 3-hydroxybenzaldehyde, trans-cinnamate, estradiol-17α, deoxycholate, retinoate, oleic acid, and heptadecanoate. Log P values of standards were calculated using the Molinspiration Chemoinformatics calculator.

The combination of log P and molecular mass were used to calculate predicted time of maximal plasma absorption (T_max_) in humans using the PCAP model [41]. The functional fingerprints of plant extracts were generated by plotting predicted human T_max_ and peak area (relative ion count) of the phytochemicals detected by LC-MS [42].

### 2.5. Dietary Intervention Using an Animal Model

#### 2.5.1. Animals and Background Diet

The study used six female pigs (Large White × Landrace, 2.5 months old, weight ~30 kg). The pigs weighed 23.2–25.4 kg (mean 24.4 kg, standard error (SE) 3 kg) at the start of the study and 42.8–45.4 kg (mean 44.5 kg, SE 0.3 kg) on study completion five week later. The pigs were housed in individual pens for the duration of the study (12 h light/dark cycle, temperature 18–24 °C). The animals were fed a commercial background diet (Ridley AgriProducts, Melbourne, VIC, Australia) at an energy intake of 0.5 MJ digestible energy/kg body weight (BW)/day representing about 80% of usual energy intake and consumed water ad libitum. The composition of the feed includes 18% protein, 40.37% starch, 2.73% sugar, 4.9% fat, 19.35% fibre, 4.95% ash, 0.9% calcium, and 0.65% phosphorus. The study was approved by the Animal Ethics Committee of the Faculty of Veterinary and Agricultural Sciences, The University of Melbourne, Australia (approval number 1513762.1).

#### 2.5.2. Cephalic Vein Catheterisation Procedure

The cephalic veins of the animals were catheterised under general anaesthesia allowing 7-day post-surgery recovery. Pigs were injected intramuscularly with ketamine hydrochloride (10 mg/kg BW; Ketalar, Pfizer, NY, USA) mixed with xylazine (1 mg/kg BW; Rompun, Bayer, Leverkusen, Germany) to induce sedation and anesthesia. Pigs were then intubated and maintained on 1–4% isoflurane inhalation anaesthesia (Rhone Merieux, Footscray West, VIC, Australia). A silastic catheter was inserted into the external cephalic vein and advanced to the anterior vena cava via the cephalic vein; exteriorisation of the catheter in the interscapular space and storage of the catheter in a cloth pouch glued to the back of the animals was performed as described previously [48]. After catheterisation, the neck incision and exit site were irrigated with benzyl penicillin (BenPen, CSL, Parkville, VIC, Australia) and the animals were given 2 mL of 150 mg/mL of antibiotic amoxicillin (Moxylan, Jurox, Rutherford, NSW, Australia) and 2 mL of 100 mg/mL analgesic/anti-inflammatory ketoprofen (Troy Labs Pty. Ltd., Smithfield, NSW, Australia). After surgery, the animals were monitored for feeding behaviour, general disposition, and rectal temperature. Any animals with elevated temperatures (>39 °C) were given 2 mL of 150 mg/mL amoxicillin. Catheters were flushed daily with physiological saline containing 100 units/mL (U/mL) heparin.

#### 2.5.3. Experimental Design and Procedure

The study was performed in a crossover 4 × 2 factorial design with the factors being two plant extracts at four doses (including placebo control) in triplicate. The wash-out period between treatments was for a minimum of two days. To account for differences in the total phenolic contents of the plant extracts, doses of red cabbage and grape skin extracts were standardised for their total phenolic content as gallic acid equivalents (GAE). On each experiment day, the pigs received a single dose of one of two treatments: red cabbage or grape skin extracts at one of four doses: 0, 2.22, 4.44, and 11.11 mg GAE/kg BW. Considering that the grape skin extract had a higher total phenolic content compared to the red cabbage extract, the doses were selected based on previous studies of grape skin extract administered safely to mice [49,50,51]. The maximal dose of 11.11 mg GAE/kg BW corresponding to 30 mg grape skin extract/kg BW was selected for our pig study, as this dose is equivalent to the proven safe dose of 200 mg grape skin extract/kg BW in mice [52].

At 8 am on each experiment day, pigs were weighed after an overnight fast. After a baseline (0 h) blood sample, pigs were gavaged with a single dose of plant extract solids reconstituted in water to 50 mL and blood samples collected every 0.5 h for 4 h. The catheter was washed before collecting each blood sample by withdrawing 10 mL of fresh blood. A 10-mL blood sample was then collected using a syringe and immediately placed into a heparinised collection tube (BD Vacutainer^®^, BD Australia, North Ryde, NSW, Australia) and immediately placed on ice. Lastly, the cannulas were refilled with 100 U/mL heparin in saline and secured in the interscapular pouch. Plasma was obtained by withdrawing supernatants of blood centrifuged at 2000× *g* for 10 min at 4 °C, and aliquots were frozen at −20 °C until analysis. During 4 h of blood sampling period, no foods were given to pigs. After the last blood sampling, pigs were fed the background diet.

#### 2.5.4. Plasma Total Antioxidant Capacity Assay

Plasma total antioxidant capacity ex vivo and in vitro was measured using the cupric reducing antioxidant capacity (CUPRAC) assay [53] and reported as Trolox equivalent antioxidant capacity (TEAC). Plasma TEAC ex vivo was performed on plasma samples collected from the pigs after oral intake of the plant extracts. Plasma TEAC in vitro was performed on reconstituted commercial pig plasma (3.8% trisodium citrate as anticoagulant, Sigma-Aldrich, St. Louis, MO, USA). Freeze-dried commercial pig plasma was reconstituted in Milli Q water to the indicated volume by the manufacture, and aliquots were frozen until analysis. On the day of plasma TEAC in vitro analysis, commercial pig plasma aliquots were thawed and spiked with either gallic acid standard, red cabbage, or grape skin extracts to final concentrations of 0.05, 0.1, 0.2, 0.4, and 0.5 mg GAE/mL. Plasma samples (collected from the pigs or spiked commercial plasma) were diluted 1:5 with Tris-glycine-urea buffer pH 7 before the CUPRAC assay.

The CUPRAC assay is based on the capacity of a sample to reduce a Cu(II) complex to a Cu(I) complex, which can be measured at 485 nm wavelength. Equal volumes (50 µL) of 7.5 mM BCS, 10 mM CuCl_2_ and Tris-glycine-urea buffer were added to each well of a 96-well plate, followed by addition of 100 µL of samples (blank, standard, or diluted plasma). The plate was incubated at 22 °C for 1 h and absorbance at 485 nm was measured. Results were reported as TEAC based on a 6-point calibration curve using Trolox as the standard (0–100 µM) after blank subtraction. Analysis was performed in duplicate. Yields of increased plasma TEAC in vitro (nmol/µmol GAE) by the spiked phytochemicals were reported as the slope of linear regression of plasma TEAC as a function of phytochemical concentrations. 

#### 2.5.5. Plasma Glutathione Peroxidase Activity

Plasma GPx activity ex vivo was performed on plasma samples collected from the pigs after oral intake of the plant extracts using a commercial kit (Trevigen, Gaithersburg, MD, USA). Briefly, plasma samples (20 µL) were added to a reaction mixture containing premixed glutathione, reduced form of nicotiamide adenine dinucleotide phosphate (NADPH), glutathione reductase, and cumene hydroperoxide. Absorbance at 340 nm were monitored at 1 min intervals for 15 min, at 25 °C. The GPx activity was calculated from the rate of change in absorbance using GPx standard as a positive control. Results were reported as units/mL, where 1 unit of GPx activity was defined as the amount of enzyme that caused the oxidation of 1 nmol of NADPH to NADP^+^ per minute at 25 °C. Analysis was performed in triplicate.

### 2.6. Hydrogen Peroxide Production of Plant Extracts in Pig Plasma In Vitro

The dose response production of H_2_O_2_ by phytochemicals in reconstituted commercial pig plasma (Sigma-Aldrich, St. Louis, MO, USA) was measured using the FOX assay [54]. Reconstituted commercial pig plasma was spiked with either gallic acid standard, red cabbage, or grape skin extracts to final concentrations of 0.05, 0.1, 0.2, 0.4, and 0.5 mg GAE/mL and was incubated at 37 °C for 1 h before the FOX assay of H_2_O_2_. The concentrations were selected to be in the equivalent range of the doses used in the animal study with pigs having 70 mL circulating blood/kg BW and plasma making up 55% of blood volume [55].

After incubation, the plasma sample was diluted 1:5 with Tris-glycine-urea buffer pH 7 and assays were conducted as follows. 90 µL of samples (blank, standard, or diluted plasma) were mixed with 10 µL of methanol and 900 µL of FOX reagent. The mixture was vortexed for 5 s followed by incubation at 22 °C for 30 min. After incubation, the mixture was centrifuged at 15,000 rpm for 10 min at 22 °C and absorbance of the supernatant was measured at 560 nm. Concentrations of plasma H_2_O_2_ were calculated based on a 6-point calibration curve using H_2_O_2_ as the standard (0–90 µM) after blank subtraction. Analysis was performed in duplicate. Yields of H_2_O_2_ production in vitro (nmol/µmol GAE) by the spiked phytochemicals were reported as the slope of the linear regression of H_2_O_2_ concentration as a function of phytochemical concentrations. 

### 2.7. Data Analysis

All curve-fitting was performed using SigmaPlot for Windows Version 12.5 (Systat Software Inc., Chicago, IL, USA). The general linear model (GLM), analysis of covariance (ANCOVA), and Tukey’s test 95% confidence grouping analyses were performed in Minitab 16 statistical software (Minitab Inc., State College, PA, USA). Pearson’s correlation analysis was performed in Minitab 16 statistical software (Minitab Inc.).

## 3. Results

### 3.1. Predicted Human Absorption as Functional Fingerprints of Plant Extracts

**Figure 1:**
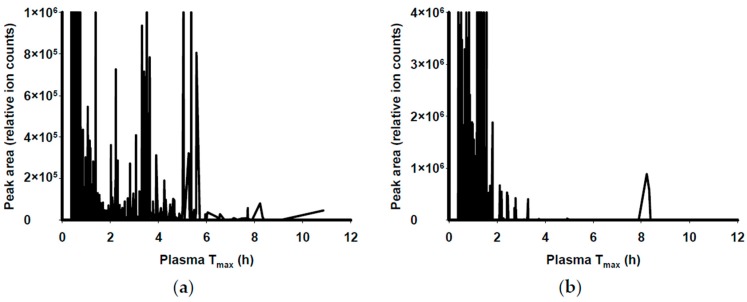
“Functional fingerprints” of plant extracts predicting absorption in humans based on the PCAP model [41] and the LC-MS method [42]. Functional fingerprints of (**a**) red cabbage; and (**b**) grape skin extracts. T_max_, the time required for phytochemicals to reach their maximal plasma concentration.

Predicted absorption as ‘functional fingerprints’ of red cabbage and grape skin extracts were analysed by our LC-MS method with the application of the PCAP model. These functional fingerprints show the predicted ranges of time required for phytochemicals in the extracts to reach their maximal plasma concentrations in human (T_max_) after oral consumption. Accordingly, red cabbage was predicted to have a long T_max_ range of 0.4–11 h (Figure 1a) whilst grape skin was predicted to have shorter T_max_ ranges of 0.4–3.7 h and 8.2–8.3 h (Figure 1b). The functional fingerprints of the plant extracts informed blood sampling time between 0–4 h at 0.5 h intervals in the current animal study.

### 3.2. Time Course Effects of Oral Consumption of Plant Extracts on Plasma Antioxidant Status Ex Vivo of Pigs

**Figure 2:**
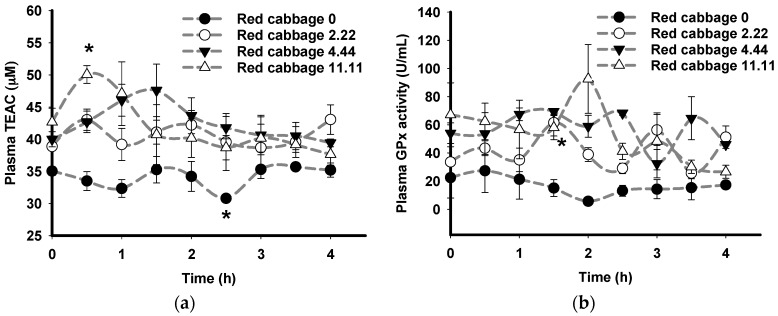
Effects of oral consumption of red cabbage extract on the plasma antioxidant status of pigs. Pigs consumed red cabbage extract at four doses in mg gallic acid equivalent/kg body weight: 0 (black circle), 2.22 (white circle), 4.44 (black triangle), and 11.11 (white triangle). Plasma antioxidant status was measured as: (**a**) plasma Trolox equivalent antioxidant capacity (TEAC); and (**b**) plasma glutathione peroxidase (GPx) activiy. Data points labelled “*” are significantly different from baseline at time 0 (*p* ≤ 0.05, Tukey’s test). Results represent the mean and error bars represent standard error of the mean (*N* = 3).

The animals consumed four doses of either red cabbage or grape skin extracts (0–11.11 mg GAE/kg BW) and plasma samples were taken every 0.5 h for 4 h. After oral consumption of red cabbage extract, in comparison to baseline at time 0, a significant increase in plasma TEAC was observed at 0.5 h in pigs consuming the maximal dose of 11.11 mg GAE/kg BW (Figure 2a) and a significant increase in plasma GPx activity was observed at 1.5 h in pigs consuming 2.22 mg GAE/kg BW (Figure 2b).

**Figure 3:**
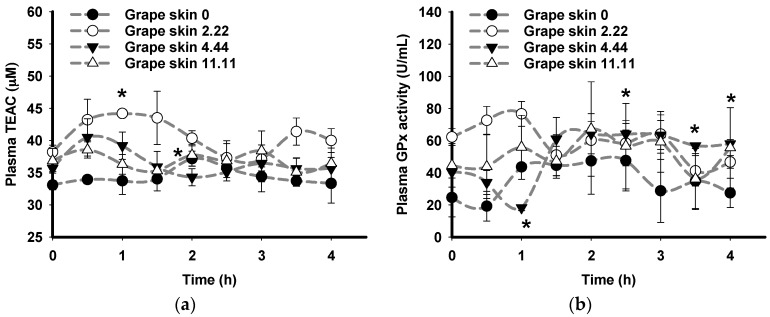
Effects of oral consumption of grape skin extract on the plasma antioxidant status of pigs. Pigs consumed grape skin extract at four doses in mg gallic acid equivalent/kg body weight: 0 (black circle), 2.22 (white circle), 4.44 (black triangle), and 11.11 (white triangle). Plasma antioxidant status was measured as: (**a**) plasma Trolox equivalent antioxidant capacity (TEAC); and (**b**) plasma glutathione peroxidase (GPx) activiy. Data points labelled “*” are significantly different from baseline at time 0 (*p* ≤ 0.05, Tukey’s test). Results represent the mean and error bars represent standard error of the mean (*N* = 3).

After consumption of grape skin extract, in comparison to baseline at time 0, a significant increase in plasma TEAC was observed after 1 h in pigs consuming 2.22 mg GAE/kg BW (Figure 3a) and significant increases of plasma GPx activity were observed at 2.5, 3.5, and 4 h in in pigs consuming 4.44 mg GAE/kg BW (Figure 3b). In contrast, a significant reduction in plasma GPx activity was observed at 1 h in pigs consuming 4.44 mg GAE/kg BW (Figure 3b).

### 3.3. Effects of Plant Extracts on Plasma Total Antioxidant Capacity and Plasma Hydrogen Peroxide Concentration In Vitro

The dose response effects of the plant extracts on plasma TEAC and plasma H_2_O_2_ concentration in vitro were analysed after spiking plasma with either gallic acid standard, red cabbage, or grape skin extracts to final concentrations of 0.05, 0.1, 0.2, 0.4, and 0.5 mg GAE/mL. Proportional increase in plasma TEAC was observed with increased concentrations of all three phytochemical sources and followed linear regression relationships (Table 1). Yields of increased plasma TEAC in vitro by the phytochemicals were 1606.3 ± 98.1, 633.2 ± 74.7, and 1077.8 ± 120.4 nmol/µmol GAE for gallic acid standard, red cabbage, and grape skin extracts, respectively (Table 1). 

Similar to plasma TEAC in vitro, proportional increase in plasma H_2_O_2_ concentrations was observed with increased concentrations of all three phytochemical sources and followed linear regression relationships (Table 1). Yields of H_2_O_2_ production in vitro by the phytochemicals in plasma were 68.7 ± 4.5, 22.4 ± 1.1, and 44.2 ± 2.1 nmol/µmol GAE for gallic acid standard, red cabbage, and grape skin extracts, respectively (Table 1).

Comparing the three phytochemical sources, significant differences in yields of plasma TEAC and plasma H_2_O_2_ were observed (*p* ≤ 0.05, ANCOVA). Further, significantly high correlation between yields of plasma TEAC and plasma H_2_O_2_ were observed (*r* = 1, *p* ≤ 0.05, Pearson’s correlation analysis), with gallic acid having the strongest effect (highest yields) followed by grape skin and red cabbage extracts (Table 1).

### 3.4. Effects of Phytochemical Dose and Their H_2_O_2_ Production Capacity In Vitro on Plasma Antioxidant Status of Pigs Ex Vivo

**Figure 4:**
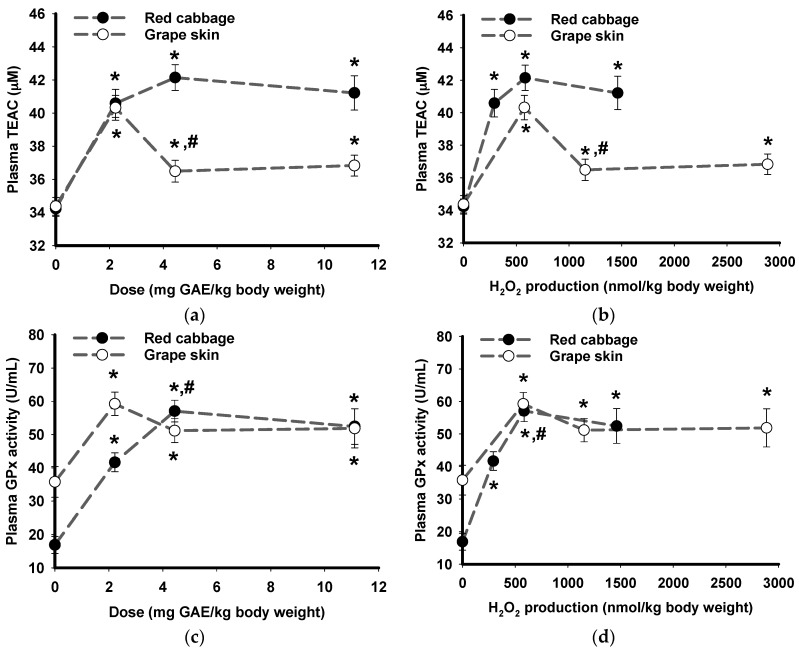
Total plasma antioxidant capacity and glutathione peroxidase activity of pig plasma as a function of phytochemical dose and H_2_O_2_ production efficacy. Means across all pig plasma sampling time points (0.5 h interval for 4 h) of plasma TEAC versus (**a**) phytochemical doses and (**b**) H_2_O_2_ production efficacy. Means across all pig plasma sampling time points of plasma GPx activity versus (**c**) phytochemical doses and (**d**) H_2_O_2_ production efficacy. The H_2_O_2_ production (nmol/kg body weight) was calculated based on the yield of H_2_O_2_ production (nmol/µmol GAE) of the plant extracts in vitro (Table 1). Data points labelled “*” are significantly different from dose 0 (*p* ≤ 0.05, Tukey’s test). Data points labelled “#” are significantly different from the previous dose (*p* ≤ 0.05, Tukey’s test). Results represent the mean and error bars represent standard error of the mean (*N* = 27).

Means across all pig plasma sampling points (0.5 h interval for 4 h, Figure 2 and Figure 3) were combined to investigate the overall dose effects of the plant extracts on pig plasma antioxidant status (Figure 4). For both plant extracts, plasma TEAC ex vivo significantly increased at all three doses 2.22, 4.44, and 11.11 mg GAE/kg BW compared to dose 0 (Figure 4a). There was no significant difference in plasma TEAC among the three doses of red cabbage extract whilst plasma TEAC at grape skin extract dose of 4.44 and 11.11 mg GAE/kg BW was significantly reduced compared to the 2.22 mg GAE/kg BW dose (Figure 4a). The phytochemical dose (mg GAE/kg BW) of the two plant extracts was standardised to their in vitro H_2_O_2_ production yields (nmol/µmol GAE, Table 1) to estimate the H_2_O_2_ production (nmol/kg BW) by the plant extract dose used in the animal study. The in vitro H_2_O_2_ production yields of the two plant extracts had similar effects on the mean plasma TEAC of pigs compared to their phytochemical dose (Figure 4b).

Similarly, for both plant extracts, significant increases in pig plasma GPx activity were observed at all three doses (Figure 4c). There was no significant difference in GPx activity among the three doses of grape skin extract whilst GPx activity at a red cabbage extract dose of 4.44 mg GAE/kg BW was significantly increased compared to the 2.22 mg GAE/kg BW (Figure 4c). After standardisation of the phytochemical dose to their in vitro H_2_O_2_ production yields (Table 1), the plasma GPx activity in response to the two plant extracts was remarkably similar (Figure 4d).

## 4. Discussion

This study examines the consumption of dietary phytochemicals by pigs and shows that non-enzymatic and enzymatic antioxidant defences were increased. Absorption kinetics of red cabbage and grape skin extracts were characterised in pigs after oral consumption using plasma TEAC as a measure of the non-enzymatic antioxidant response [56] and plasma GPx (an antioxidant enzyme) activity [57]. The blood sampling time of the study (0.5 h interval for 4 hours) was chosen to capture the range of time expected for the phytochemicals to achieve their maximal plasma concentrations (T_max_), predicted from their functional fingerprints (0.4–4 h). Consistent with the predicted functional fingerprints, a significant increase in plasma TEAC was observed at 0.5 h after consumption of red cabbage (11.11 mg GAE/kg BW) and at 1 h after consumption of grape skin (2.22 mg GAE/kg BW). Peaks of plasma TEAC have been observed to coincide with peaks of plasma phytochemicals in humans after consumption of tea [58] and chocolate [59]. Therefore, the identification of increased plasma TEAC within this selected time frame after plant extract ingestion validates the utility of the phytochemical absorption prediction (PCAP) model [41] and its application to the production of the functional fingerprints. 

These results highlight the ability of the PCAP model to guide experimental design to ensure that the functional impact of the phytochemicals is captured during the sampling regime. For example, a previous study investigating the pharmacokinetics of three phytochemicals carvacrol, thymol, and eugenol in pigs reported the time of maximal absorption (T_max_) at 1.39, 1.35, and 0.83 h, respectively [60]. Using our PCAP model [41], the T_max_ of these phytochemicals for humans was predicted to be 1.76, 1.67, and 1.58 h, respectively. Comparing to the reported T_max_ in pigs [60], the predicted T_max_ of these phytochemicals in humans was very similar and followed the same sequence with T_max_ of carvacrol > thymol > eugenol. This similarity of observed T_max_ compared to predicted T_max_ suggests that the PCAP model can be useful for predicting absorption of phytochemicals in pigs as well as in humans.

In the present study, the plant extract doses were standardised for their respective total phenolic contents as GAE analysed by the Folin-Ciocateu assay. Whilst ascorbic acid is known to interfere with this assay [61], based on analyses conducted by others [61,62,63,64,65,66,67], the contributions of ascorbic acid to the GAE results are estimated to be 0.3% and 4% for grape skin and red cabbage extracts, respectively. These minimal contributions of ascorbic acid to the GAE results reflected the naturally low ascorbic acid content of grape skin [62], and the effects of microwave cooking [66] and ultrasonication [67] which reduced the ascorbic acid content of red cabbage during plant processing. Accordingly, the GAE results presented herein are considered accurate indicators of the total phenolic content of the two plant extracts.

In comparison to plasma TEAC, a delayed increase in plasma GPx activity was observed at 1.5 h after consumption of red cabbage (2.22 mg GAE/kg BW) and at 2.5, 3.5, and 4 h after consumption of grape skin (4.44 mg GAE/kg BW). The observed time delay of plasma GPx activity after plasma TEAC is consistent with a previous study [68]. This delay may be explained by the induction of GPx activity occurring in response to the presence of phytochemicals in the plasma, as indicated by increased plasma TEAC [58,59]. Accordingly, increased plasma TEAC and increased plasma GPx activity after consumption of the plant extracts indicate that phytochemicals provide health benefits via both direct antioxidant activity and indirectly via the induction of enzymatic antioxidant defence mechanisms.

The dose response effects of red cabbage and grape skin extracts increased plasma TEAC in vitro after direct addition of the extracts to the pig plasma in the present study. As the phytochemical doses increased, there was a proportional increase in plasma TEAC in vitro (633.2–1606.3 nmol/µmol GAE), supporting the direct antioxidant activity of phytochemicals in vitro as observed in many studies [69,70,71]. In comparison to the in vitro experiments, same doses of red cabbage and grape skin extracts consumed by the pigs did not result in a proportional increase in plasma TEAC and plasma GPx activity ex vivo. When plant extracts were orally administered to pigs, increased plasma TEAC was observed at all doses compared to 0 but an increase in dose did not result in significant further increase of TEAC above the lowest dose. Further, an increase in dose of grape skin extract resulted in decreased plasma TEAC at doses of 4.44 and 11.11 mg GAE/kg BW compared to the dose at 2.22 mg/kg BW. The differences in plasma TEAC responses to the plant extracts may be attributed to their distinct phytochemical compositions [44,46]. Similarly, plasma GPx activity significantly increased for all doses compared to dose 0 but further increase of doses did not show a clear response relationship. 

The observed differences between in vitro and ex vivo have also been observed in other studies [69,70]. Direct addition of tea [69] or apple phytochemicals [70] to human plasma in vitro increased plasma TEAC. However, consumption of the same or higher concentrations of tea [69] and apple phytochemicals [70] by humans did not reproduce the same effects as observed in vitro. The differences between in vitro and ex vivo results can be explained by the low bioavailability of phytochemicals in vivo as they are handled by the body as xenobiotics [40]. Further, these differences may be attributed to the increased complexity of the in vivo system wherein both direct and indirect antioxidant mechanisms may arise, as indicated by increased plasma GPx activity ex vivo.

Hypothetical pro-oxidant effects of phytochemicals in vitro via measurement of H_2_O_2_ levels in plasma were studied after direct addition of plant extracts. Similar to the results measuring plasma TEAC in vitro, incubation of red cabbage and grape skin extracts in pig plasma resulted in a proportional increase in plasma H_2_O_2_ levels (22.4–68.7 nmol/µmol GAE). Pro-oxidant effects of phytochemicals in vitro have been observed in the presence of oxygen and metal ions such as copper and iron [35,36,37,72,73,74,75]. Concentrations of iron and copper ions in human plasma are 2.13 and 0.81 µg/g, respectively [76], and iron levels of 0.1 µg/g [35] and copper levels of 3 µg/g [73] have been reported to initiate H_2_O_2_ production in vitro. The formation of H_2_O_2_ by phytochemicals in plasma observed here may be attributed to the electron transfer process between phytochemicals, oxygen, and metal ions present in plasma [73].

The ability of phytochemicals to produce H_2_O_2_ has been proposed to be responsible for the cytotoxic effects of phytochemicals in cell culture studies [35,36,37]. H_2_O_2_ has been widely used as an oxidative stress inducer in many studies investigating the protective effects of phytochemicals in response to oxidative stress [77,78,79]. However, the ability of phytochemicals to produce H_2_O_2_ may explain their indirect antioxidant protection mechanism. High concentrations of H_2_O_2_ (≥100 µM) are harmful for cells but low concentrations of H_2_O_2_ (≤50 µM) can be beneficial to initiate the antioxidant cellular defence [80,81]. Low concentrations of H_2_O_2_ have been observed to stimulate wound healing in keratinocytes [82] and in mice [83]. Similarly, the health benefits of regular exercise have been proposed to be associated with their production of low levels of ROS (such as H_2_O_2_) that induce adaptive responses to protect against molecular damage and, subsequently, aging [84,85]. Supporting this mechanism, H_2_O_2_ has been reported to activate the nuclear factor-erythroid-2-related factor 2 (Nrf2) de novo [86] which is a transcription factor involved in inducing the antioxidant response by regulating coordinated induction of stress response genes encoding antioxidant enzymes such as superoxide dismutase, catalase, and GPx [87]. Activation of Nrf2 has been proposed as a therapeutic potential for protection against chronic diseases [87,88]. Many phytochemicals are known as Nrf2 activators including curcumin (in turmeric) [89], epigallocatechin gallate (in green tea) [90], lycopene (in tomato) [91], resveratrol (in grape) [92], and sulforaphane (in broccoli) [93]. Accordingly, H_2_O_2_-mediated induction of Nrf2 in response to phytochemical supply may explain the correlation between H_2_O_2_ production and increased plasma GPx activity observed in our study. 

## 5. Conclusions

The findings of the current study provide new insights in mechanisms by which dietary phytochemicals impact health, apart from direct ROS-scavenging pathways. An additional role is proposed whereby protection against oxidative tissue damage results from the promotion of cellular oxidative stress defence by dietary phytochemicals. This research demonstrates for the first time that H_2_O_2_ production analysis represents a useful predictive indicator of the in vivo efficacy of dietary phytochemicals.

## Figures and Tables

**Table 1:** Effects of plant extracts on plasma Trolox equivalent antioxidant capacity (TEAC) and plasma levels of hydrogen peroxide (H_2_O_2_) in vitro.

Phytochemical Sources	Plasma TEAC		Plasma H_2_O_2_	
	Yield (nmol/µmol GAE) *	Linear Fit *R^2^*	Yield (nmol/µmol GAE) *	Linear Fit *R*^2^
Gallic acid standard	1606.3 ± 98.1	0.99	68.7 ± 4.5	0.97
Red cabbage extract	633.2 ± 74.7	0.96	22.4 ± 1.1	0.99
Grape skin extract	1077.8 ± 120.4	0.96	44.2 ± 2.1	0.99

* Gallic acid standard and plant extracts were directly spiked into commercial pig plasma at concentrations of 0.05–0.5 mg gallic acid equivalent (GAE)/mL. Increased plasma TEAC and plasma H_2_O_2_ levels followed linear regressions with slopes representing yields of increase. Comparing three phytochemical sources, significant differences in yields of plasma TEAC and plasma H_2_O_2_ were observed (*p* ≤ 0.05, analysis of covariance (ANCOVA)). Significantly high correlation between plasma TEAC and plasma H_2_O_2_ was observed (*r* = 1, *p* ≤ 0.05, Pearson’s correlation analysis). Results represent the mean ± standard error of the mean (*N* = 2).
